# Cutoff Values for Screening Post-Intensive Care Syndrome Using the Post-Intensive Care Syndrome Questionnaire

**DOI:** 10.3390/jcm14113897

**Published:** 2025-06-01

**Authors:** Jiwon Hong, Jiyeon Kang

**Affiliations:** 1 Department of Nursing, Youngsan University, Yangsan-si 50510, Republic of Korea; 2 College of Nursing, Dong-A University, Busan 49201, Republic of Korea

**Keywords:** intensive care units, ROC curve, sensitivity and specificity, survivors

## Abstract

**Background**: Post-intensive care syndrome (PICS) affects over half of intensive care unit (ICU) survivors, impairing their long-term health and quality of life. Although the Post-Intensive Care Syndrome Questionnaire (PICSQ) was developed to measure PICS, validated cutoff values for screening are lacking. This study aimed to determine optimal cutoff values for each domain of the PICSQ. **Methods**: A total of 475 ICU survivors completed the PICSQ three months after discharge. Receiver operating characteristic (ROC) curve analyses were conducted to determine optimal cutoff values for each domain. The criterion tools included the Hospital Anxiety and Depression Scale, the Posttraumatic Diagnostic Scale, the Activities of Daily Living scale, and the Montreal Cognitive Assessment. Health-related quality of life and hospital readmission rates were compared between groups classified by the determined cutoffs. **Results**: The optimal cutoff values were ≥3 for mental, ≥7 for physical, and ≥2 for cognitive domains, with area under the curve (AUC) values of 0.83, 0.84, and 0.80, respectively. The participants scoring above these cutoffs had significantly lower quality of life and higher readmission rates. **Conclusions**: The determined cutoff values may support early screening of PICS in ICU survivors, enabling timely interventions to improve long-term outcomes. Further research is needed to validate these values in diverse populations.

## 1. Introduction

Approximately 90% of patients admitted to the intensive care unit (ICU) now survive [1,2]. However, survival is often followed by new challenges. Over half of ICU survivors experience post-intensive care syndrome (PICS), a condition characterized by new or worsening mental, physical, and cognitive impairments following ICU treatment [3]. In the past decade since the syndrome was first described, research has expanded to encompass its prevalence, risk factors, measurement tools [4,5], and long-term outcomes [6]. PICS not only negatively affects long-term outcomes in ICU survivors but also causes psychological burden and mental health problems in their families [6,7]. Despite the growing awareness of PICS, effective tools for early screening in clinical settings remain limited.

PICS is not a medical diagnosis but rather a set of impairments that ICU survivors may experience during their recovery [8]. As a result, practical and easy-to-use tools are needed to monitor these impairments in clinical settings. To measure PICS in ICU survivors, various tools have been proposed to assess key problems within its three domains, such as depression, anxiety, post-traumatic stress disorder (PTSD), physical dysfunction, and mild cognitive impairment [9,10]. However, assessing each of the three domains of PICS with these tools has the disadvantage of being time-consuming and requiring specialized training or equipment, making it difficult to apply in routine clinical practice. To address this, the Post-Intensive Care Syndrome Questionnaire (PICSQ), an 18-item self-report scale, was developed to comprehensively measure three PICS domains—mental, physical, and cognitive impairments—in ICU survivors [11]. Because of its concise format and minimal constraints, it may serve as a practical tool in busy clinical settings.

To effectively utilize the newly developed PICSQ in research and clinical practice, determining optimal cutoff values for each domain is important. Appropriate cutoff values enable healthcare professionals to establish timely intervention plans through risk screening. In other words, an appropriate cutoff value can improve the utility of the tool. Recently, cutoff values for the PICSQ to screen for the risk of unplanned readmission have been reported [12]. However, these cutoff values are limited in their scope because they only address unplanned readmissions. The importance of a multidisciplinary approach to the assessment and management of PICS has been increasingly emphasized [13], and the Society of Critical Care Medicine (SCCM) is currently developing guidelines for both PICS and PICS-family [14]. In line with these global trends, establishing optimal cutoff values for efficient screening using the PICSQ may contribute to improving the long-term outcomes of ICU survivors. Therefore, this study aims to determine domain-specific cutoff values for the PICSQ to facilitate effective screening of PICS in ICU survivors.

## 2. Materials and Methods

### 2.1. Design

This study was a methodological secondary analysis that aimed to determine optimal cutoff values for each domain of the PICSQ. The original study examined the incidence and risk factors of PICS subtypes among ICU survivors three months after discharge [4].

### 2.2. Setting and Participants

The participants in the primary study were patients aged 18 years or older who had been admitted for more than 24 h in 19 ICUs at four university hospitals in Busan, Korea. Patients were excluded if they had been readmitted to the ICU within the past year or had a pre-existing diagnosis of mental, physical, or cognitive impairments before ICU admission. In addition, patients were excluded if they were unable to complete the survey due to communication limitations. These limitations included cases in which patients themselves reported difficulty communicating because of language disorders, ventilator dependence, or tracheostomy.

We analyzed data from 475 of the 891 cohort participants who responded to a follow-up survey three months after discharge, as the impact of critical illness and ICU treatment is most severe in the early recovery period of 3–6 months [15]. Before conducting the analysis, we calculated the required sample size for the two primary statistical methods to ensure that the original data were sufficient. For the receiver operating characteristic (ROC) curve analysis, using MedCalc statistical software (version 19.2.0; MedCalc Software Ltd., Ostend, Belgium) with a significance level of 0.05, power of 0.80, and an area under the ROC curve (AUC) of 0.70 [12], the minimum required sample size was 62. For group comparison analysis, assuming an effect size of 0.29 [16,17], a significance level of 0.05, and a power of 0.80, the minimum required sample size for an independent *t*-test was 376. Therefore, the sample size in this study was sufficient for both types of analyses.

### 2.3. Measurements

#### 2.3.1. The Post-Intensive Care Syndrome Questionnaire

The PICSQ is a self-report measurement tool that assesses all three domains of PICS: mental, physical, and cognitive impairment [11]. In this study, we used the validated Korean version of the PICSQ, which consists of 18 items, with 6 items in each domain. The respondents rated items on a 4-point Likert scale (from 0 = never to 3 = always), focusing on impairments that were new or worsened after ICU treatment compared to before admission. The possible score range for each area was 0 to 18. The Cronbach’s α reliability coefficients reported at the time of the PICSQ’s development ranged from 0.84 to 0.90 [11], and in this study, they were 0.83 for the mental domain, 0.83 for the physical domain, and 0.85 for the cognitive domain.

#### 2.3.2. Criterion Tools for PICS

The participants were considered to have PICS in the mental domain if they screened positive for one of the following: anxiety, depression, or PTSD. We assessed anxiety and depression using the Korean version of the Hospital Anxiety and Depression Scale (K-HADS). The K-HADS comprises 14 items—7 for anxiety and 7 for depression—rated on a 4-point Likert scale. Total scores range from 0 to 21 for each subscale, with scores ≥8 indicating positive anxiety or depression [18]. In this study, the Cronbach’s α reliability coefficients were 0.85 for HADS-Anxiety and 0.89 for HADS-Depression. To measure PTSD, we used the Posttraumatic Diagnostic Scale (PDS), which assesses PTSD symptoms based on criteria from the Diagnostic and Statistical Manual of Mental Disorders, Fourth Edition [19,20]. The PDS consists of 17 items rated on a 4-point Likert scale, with total scores ranging from 0 to 51. A score ≥11 indicates positive PTSD [19,21]. The Cronbach’s α reliability coefficient for the PDS in this study was 0.85.

The physical domain of PICS was assessed using the Korean Activities of Daily Living (K-ADL) scale, which is based on the Katz scale [22]. The K-ADL scale consists of seven items evaluating basic activities of daily living, with total scores ranging from 7 to 21. A score ≥8 indicates non-independence in daily activities [23], which we considered indicative of physical PICS. The Cronbach’s α for this scale in our study was 0.93.

The cognitive domain of PICS was evaluated using either the Montreal Cognitive Assessment (MoCA) or the MoCA-BLIND for telephone interviews when necessary. The MoCA comprises 32 items assessing various cognitive functions, including short-term memory, visuospatial abilities, executive functions, attention, concentration, working memory, language, and orientation to time and place. MoCA scores range from 0 to 30. The MoCA-BLIND, a modified version for remote assessment, consists of 24 items from the original MoCA, excluding those requiring face-to-face evaluation. MoCA-BLIND scores range from 0 to 22 [24]. We converted MoCA-BLIND scores to equivalent MoCA scores using the official conversion formula (https://www.mocatest.org/). A cutoff score of ≤22 was used to indicate mild cognitive impairment [25], which we considered representative of cognitive PICS.

#### 2.3.3. Tools for Validating Cutoff Values

We compared hospital readmission rates and health-related quality of life (HRQoL) between PICS and non-PICS groups to validate the cutoff values. Readmissions were self-reported by participants. We assessed HRQoL using the Medical Outcomes Study Short Form version 2 (SF-36v2), which comprises 36 items evaluating 8 domains. These domains are psychometrically combined to derive a physical component summary (PCS) and a mental component summary (MCS). The PCS encompasses physical functioning, role-physical, bodily pain, and general health, while the MCS includes vitality, social functioning, role-emotion, and mental health [26]. We utilized the PROCoRE Version 2.1 smart measurement system (QualityMetric Inc., Johnston, RI, USA) to calculate scores. The scores are normalized against HRQoL norms for healthy adults in the United States (mean 50, standard deviation 10), with a possible range of 0–100.

### 2.4. Data Collection

The primary cohort data used in this study were collected between 1 June 2019 and 31 July 2020. The participants were recruited publicly through recruitment notices and information leaflets. The participants were provided with sufficient information about the study and voluntarily signed a written informed consent form if they agreed to participate (see Institutional Review Board Statement and Informed Consent Statement for details). Five trained research assistants conducted the surveys following an established protocol. To ensure consistency, the research assistants participated in regular seminars and training sessions. Initially, surveys were conducted face-to-face. However, due to the COVID-19 pandemic, the method was changed to non-face-to-face surveys via phone calls. Survey completion time varied by participant but typically took 20 to 30 min.

### 2.5. Statistical Analysis

We analyzed data using MedCalc statistical software (version 19.2.0; MedCalc Software Ltd., Ostend, Belgium) and IBM SPSS Statistics (version 25.0; IBM Inc., Armonk, NY, USA). Demographic and clinical characteristics of the participants were analyzed using descriptive statistics. To determine the optimal cutoff values, we conducted a ROC analysis. An AUC value of 0.50 or higher indicates discriminatory ability, with values above 0.70 suggesting moderate accuracy and those exceeding 0.90 demonstrating high accuracy [27,28]. We identified cutoff values for each PICSQ domain by maximizing the Youden index (*J* = Sensitivity + Specificity − 1). In selecting these cutoff values, we ensured that the positive likelihood ratio was 3 or higher for each domain [29]. Differences in HRQoL and hospital readmission rates between the PICS and non-PICS groups were analyzed using independent *t*-tests and χ^2^-tests, respectively. In this study, data with missing responses were excluded from the analysis.

## 3. Results

### 3.1. Participant’s Characteristics

The average age of the participants was 60.48 ± 13.12 years, and the majority (59.4%) were male. Most admissions (78.5%) were planned, and nearly half (49.3%) of the patients were admitted to the ICU via the emergency department. The most common reasons for ICU admission were postoperative management (30.7%), neurological disease (24.8%), and cardiovascular disease (20.8%). The most prevalent ICU type was surgical (42.1%). Illness severity was measured using the Acute Physiology and Chronic Health Evaluation II (APACHE II) and Simplified Acute Physiology Score II (SAPS II), with average scores of 11.51 ± 6.02 and 33.16 ± 15.36, respectively. The average length of ICU stay was 4.63 ± 7.90 days, and most participants (85.7%) were discharged home (Table 1).

### 3.2. Optimal Cutoff Values for Each Domain of PICSQ

The ROC analysis showed an AUC of 0.83, indicating moderate accuracy for the mental domain of PICSQ. The optimal cutoff value with the highest Youden index was 3, with a sensitivity of 67.3%, specificity of 84.9%, and positive likelihood ratio of 4.47. Participants scoring ≥3 can be considered as having PICS in the mental domain.

For the physical domain of PICSQ, the AUC was 0.84, also suggesting moderate accuracy. At the optimal cutoff value of 7, sensitivity was 68.5%, specificity was 81.9%, and the positive likelihood ratio was 3.78. Participants scoring ≥7 can be considered as having PICS in the physical domain.

**Figure 1:**
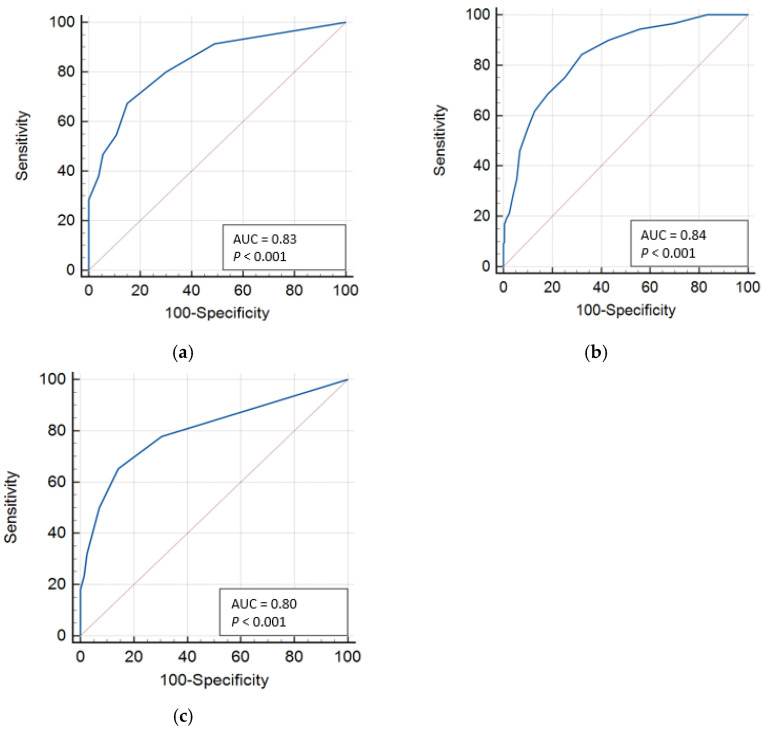
ROC curve of PICSQ domain: (**a**) mental domain; (**b**) physical domain; (**c**) cognitive domain. Abbreviations: AUC = area under the ROC curve; PICSQ = post-intensive care syndrome questionnaire; ROC = receiver operating characteristic.

Regarding the cognitive domain, the ROC analysis showed an AUC of 0.80, indicating moderate accuracy. The optimal cutoff value with the highest Youden index was 2, with a sensitivity of 65.3%, specificity of 85.7%, and positive likelihood ratio of 4.55. Participants scoring ≥2 can be considered as having PICS in the cognitive area (Table 2, Figure 1).

### 3.3. Comparison of HRQoL and Readmission Rates Between PICS and Non-PICS Groups

The participants were divided into PICS and non-PICS groups based on cutoff values determined through the ROC analysis, and their HRQoL and hospital readmission rates were compared (Table 3). Of the participants, 409 provided data on HRQoL and readmission rates. In the PICSQ, the participants were classified as having PICS if they scored ≥3 in the mental domain, ≥7 in the physical domain, or ≥2 in the cognitive domain.

Compared to their respective non-PICS group, the participants in the mental PICS group showed significantly lower HRQoL scores in both PCS (t = 8.15, *p* < 0.001) and MCS (t = 10.71, *p* < 0.001), as well as a significantly higher hospital readmission rate (χ^2^ = 7.50, *p* = 0.006). Similarly, the physical PICS group demonstrated significantly lower HRQoL scores in both PCS (t = 14.43, *p* < 0.001) and MCS (t = 5.95, *p* < 0.001), and a significantly higher hospital readmission rate (χ^2^ = 10.69, *p* = 0.001). In the cognitive PICS, PCS (t = 7.78, *p* < 0.001) and MCS (t = 5.03, *p* < 0.001) were also significantly lower, and readmission rates were significantly higher (χ^2^ = 9.02, *p* = 0.003) compared to the non-PICS group for the cognitive domain.

## 4. Discussion

Using ROC analysis with data from 475 ICU survivors, we determined the optimal cutoff values for the PICSQ to be 3 for the mental domain, 7 for the physical domain, and 2 for the cognitive domain, which demonstrated acceptable discriminatory power with an AUC above 0.80. The PICS group, classified using these cutoff values, had significantly higher hospital readmission rates and lower HRQoL than the non-PICS group.

Although the reliability and validity of the PICSQ have been established in previous studies [11], optimal cutoff values for clinical screening had not been proposed. Our findings contribute to the utility of the PICSQ by providing empirically derived cutoff values that may facilitate early screening of survivors at PICS risk. The AUC for each PICSQ domain exceeded 0.80 in our study. The AUC reflects a test’s overall ability to discriminate between the presence and absence of a condition [27,28]. An AUC closer to 1 indicates better overall performance, while those closer to 0.5 suggest poorer performance [28]. Although there’s no universally accepted standard for interpreting the AUC, scores between 0.70 and 0.90 typically are generally considered to indicate moderate accuracy, while values above 0.90 suggest high accuracy [27,28]. In this study, the PICSQ demonstrated moderate accuracy in screening the presence or absence of mental, physical, and cognitive PICS. Furthermore, the PICSQ’s AUC values were comparable to those of established PICS reference tools [30,31,32]. These results support its clinical validity in screening meaningful PICS problems.

We determined the cutoff values by considering the Youden index for a balance between sensitivity and specificity. The Youden index is commonly used to determine cutoff values that maximize the combined performance of sensitivity and specificity. However, depending on the intended purpose of the tool, cutoff values that prioritize either sensitivity or specificity may be more appropriate. For instance, highly sensitive tests are crucial for highly contagious diseases or those with high mortality rates but clear treatment options [28]. Conversely, highly specific tests are preferable when misdiagnosis carries significant psychological risks or when screening large populations [28,33]. PICS is a concept highlighting the multifaceted impairments that may occur after intensive care, rather than a specific medical diagnosis [8]. Consequently, PICS assessment tools should prioritize the screening and follow-up of survivors with potential issues, necessitating high specificity. The optimal cutoff values for each PICSQ domain in this study are well-suited for PICS screening, as they exhibit both a high Youden index and high specificity.

Given the inverse relationship between sensitivity and specificity, the final PICSQ cutoff values were associated with relatively lower sensitivity. Measurement tools with low sensitivity carry a higher risk of false-negative results. To address this, we considered the positive likelihood ratio [29], which indicates how much more likely the case group is to receive a positive result compared to the normal group. In our study, the positive likelihood ratio of PICSQ ranged from 3.78 to 4.55, indicating that the PICS group was 3–4 times more likely to test positive than the normal group based on the cutoff value. This suggests that despite its moderate sensitivity (65.3–68.5%), PICSQ demonstrates the ability to effectively screen for the PICS group. For tests with low sensitivity, repeated testing at short intervals can help reduce false-negative results [33]. Therefore, periodic administration of the brief and simple PICSQ during follow-up care of ICU survivors post-discharge may compensate for the tool’s limited sensitivity.

HRQoL and readmission rates are critical long-term outcome measures for ICU survivors [34,35]. Our study found that the PICS group, classified by the PICSQ, had significantly lower HRQoL than the normal group, with scores approximately 5–14 points below normative values [34]. PICS is also a known major risk factor for early readmission [36]. The readmission rate within three months of discharge in the PICS group, as classified by our determined cutoff values, was significantly higher than in the normal group. These findings indicate that the PICSQ’s cutoff values for the three domains effectively screened for the PICS group, validating the tool’s ability to screen at-risk individuals.

Early screening for PICS is essential to improve the HRQoL of ICU survivors [13]. The PICSQ is a convenient clinical tool that assesses all three PICS domains through 18 self-report items. It requires no specialized training and can be easily administered by any healthcare provider. Based on PICSQ scores, clinicians can efficiently screen individuals at risk and initiate tailored interventions. For example, if a patient scores ≥3 in the mental domain, clinicians may provide psychological education, recommend therapy, or use ICU diaries to support recovery. For patients scoring ≥7 in the physical domain, early mobilization or strength training could be considered depending on the patient’s condition. A score ≥2 in the cognitive domain may prompt interventions such as stress reduction, communication support, or family involvement. Although the PICSQ is intended as a screening tool and should be followed by comprehensive assessments when impairments are suspected, it serves a critical role in bridging the gap between initial detection and timely intervention. PICS rehabilitation should be delivered across a continuum of care, extending from the ICU to inpatient and outpatient settings, and eventually into the community and home [13]. There has been growing interest in remote ICU follow-up models as part of this care continuum [37]. To support this wide range of care environments, screening tools must be both accessible and adaptable. The PICSQ may also serve as a practical screening tool in telehealth settings. Its validated cutoff values enable early identification of at-risk patients, even in settings with limited clinical resources, and may ultimately contribute to improved long-term outcomes for ICU survivors.

This study has several limitations. The primary data were collected from a single region in Korea, a country with a single-payer national health system but largely private provision of healthcare. The study included only ICU survivors who were available for follow-up after discharge. These characteristics of the study population and setting may limit the generalizability of the findings. However, the demographic and clinical characteristics of the participants were comparable not only to those reported in a national cohort of ICU survivors based on Korean National Health Insurance data [38] but also to those described in studies using two large U.S. databases (MIMIC-III and eICU) and a U.K. dataset (PIRAMID) [39], suggesting that sampling bias likely did not substantially affect the findings. This study analyzed data collected at three months post-discharge, a period during which the impact of critical illness and ICU treatment is often most pronounced [15]. However, because PICS symptoms can persist for years, future research should examine long-term trajectories and outcomes. Additionally, the relatively low sensitivity observed across all three domains indicates that some individuals at risk for PICS may not be screened using the current cutoff values. Therefore, further validation of the determined cutoffs in more diverse populations and across different follow-up periods is needed, as well as studies evaluating whether early screening using the PICSQ improves clinical outcomes.

## 5. Conclusions

In this study, the ROC analysis determined the optimal PICSQ cutoff values for screening PICS in ICU survivors: 3 for the mental domain, 7 for the physical domain, and 2 for the cognitive domain. These cutoff values enable the PICSQ to screen PICS with moderate accuracy. The determined cutoff values are expected to enhance the PICSQ’s clinical utility, facilitating appropriate screening and intervention for the mental, physical, and cognitive needs of ICU survivors. We recommend future studies that diversify both the post-discharge follow-up period and participant demographics to further expand the PICSQ’s clinical applicability. Furthermore, the use of a simple and validated PICS screening tool may raise awareness of PICS among healthcare professionals and promote early detection and management, ultimately contributing to improved long-term outcomes for ICU survivors.

## Figures and Tables

**Table 1:** Characteristics of participants (*N* = 475).

Variables	Categories	*n* (%)	M ± SD
Gender	Male	282 (59.4)	
	Female	193 (40.6)	
Age (years)	<60	199 (41.9)	60.48 ± 13.12
	≥60	276 (58.1)	
Awareness of ICU	Yes	373 (78.5)	
admission	No	102 (21.5)	
ICU admission route	Emergency department	234 (49.3)	
	Others	241 (50.7)	
Reasons for ICU	Postoperative monitoring	146 (30.7)	
admission	Neurologic disease	118 (24.8)	
	Cardiovascular disease	99 (20.8)	
	Other medical conditions	77 (16.3)	
	Physical injury	35 (7.4)	
Types of ICU	Surgical	200 (42.1)	
	Cardiovascular	93 (19.6)	
	Neurological	87 (18.3)	
	Medical	46 (9.7)	
	Others	49 (10.3)	
Disease severity	APACHE II	275 (57.9)	11.51 ± 6.02
at admission	SAPS III	151 (31.8)	33.16 ± 15.36
	Not recorded	49 (10.3)	
ICU length of stay	<4	306 (64.4)	4.63 ± 7.90
(days)	≥4	169 (35.6)	
Discharge place	Home	407 (85.7)	
	Others	68 (14.3)	

Abbreviations: APACHE II = acute physiology and chronic health evaluation; ICU = intensive care unit; SAPS = simplified acute physiology score.

**Table 2:** Area under the ROC curve and cutoff value of PICSQ.

Variable	AUC	95% CI	Cutoff Value	Sensitivity (%)	Specificity (%)	Youden Index (J)	LR+
PICS mental	0.83	0.80 to 0.87	≥2	80.0	69.9	0.50	2.65
			≥3	67.3	84.9	0.52	4.47
			≥4	54.7	89.2	0.44	5.08
PICS physical	0.84	0.80 to 0.87	≥6	75.3	74.8	0.50	2.99
			≥7	68.5	81.9	0.50	3.78
			≥8	61.8	87.1	0.49	4.81
PICS cognitive	0.80	0.75 to 0.84	≥1	77.8	69.5	0.47	2.55
			≥2	65.3	85.7	0.51	4.55
			≥3	50.0	92.8	0.43	6.97

Abbreviations: AUC = area under the ROC curve; CI = confidence interval; LR+ = positive likelihood ratio; PICS = post-intensive care syndrome; PICSQ = post-intensive care syndrome questionnaire; ROC = receiver operating characteristic.

**Table 3:** Comparison of health-related quality of life and readmission rate according to cutoff values for each PICSQ domain (*N* = 409).

Variables	Categories	PICS Mental			PICS Physical			PICS Cognitive		
		<3	≥3	t or χ^2^ (*p*)	<7	≥7	t or χ^2^ (*p*)	<2	≥2	t or χ^2^ (*p*)
		M ± SD or *n* (%)			M ± SD or *n* (%)			M ± SD or *n* (%)		
Health-related Quality of Life	PCS	46.24 ± 9.66	37.73 ± 9.69	8.15 (<0.001)	47.05 ± 8.90	33.64 ± 7.86	14.43 (<0.001)	45.75 ± 9.70	36.85 ± 9.83	7.78 (<0.001)
	MCS	54.71 ± 8.64	42.24 ± 11.56	10.71 (<0.001)	53.09 ± 9.56	44.67 ± 13.16	5.95 (<0.001)	52.68 ± 9.91	45.30 ± 13.16	5.03 (<0.001)
Readmission rate	Yes	52 (18.1)	37 (30.3)	7.50 (0.006)	55 (17.9)	34 (33.3)	10.69 (0.001)	58 (18.4)	31 (33.0)	9.02 (0.003)
	No	235 (81.9)	85 (69.7)		252 (82.1)	68 (66.7)		257 (81.6)	63 (67.0)	

Abbreviations: M = mean; MCS = mental component summary; PCS = physical component summary; PICS = post-intensive care syndrome; PICSQ = post-intensive care syndrome questionnaire; SD = standard deviation.

## Data Availability

The data presented in this study are available on request from the corresponding author. The data are not publicly available due to ethical restrictions and institutional policies.
